# Repurposing Antimalarials for Oral Cancer: Selective Efficacy of Hydroxychloroquine on Gingival Squamous Cell Carcinoma

**DOI:** 10.3390/ijms262210994

**Published:** 2025-11-13

**Authors:** Sana Baroudi, Diego Alejandro González Poleo, Hawraa Issa, Mikhlid H. Almutairi, Abdelhabib Semlali

**Affiliations:** 1 Groupe de Recherche en Ecologie Buccale, Faculty of Dentistry, Université Laval, Quebec City, QC G1V 0A6, Canada; 2 Department of Zoology, College of Science, King Saud University, P.O. Box 2455, Riyadh 11451, Saudi Arabia

**Keywords:** chloroquine, hydroxychloroquine, oral cancer, apoptosis, autophagy

## Abstract

Oral cancer, the most common head and neck malignancy, has a high recurrence rate and poor prognosis largely owing to chemotherapy resistance. The adverse effects of conventional therapies have prompted investigations into safer and more effective alternative therapies. Chloroquine (CQ) and hydroxychloroquine (HCQ) have shown potential owing to their roles in autophagy modulation and immune regulation. This study clarifies the selective efficacy of hydroxychloroquine (HCQ) and chloroquine (CQ) in oral squamous cell carcinoma models, emphasizing distinct responses in gingival (Ca9-22) and tongue (SCC-9) carcinoma cells. Non-oncogenic oral epithelial cells (GMSM-K) and oral carcinoma cell lines from the tongue (SCC-9, Cal-27) and gingiva (Ca9-22) were used. Cell viability, cytotoxicity, and colony formation were assessed via MTT, LDH, and crystal violet assays. Flow cytometry was used to measure apoptosis, autophagy, oxidative stress, mitochondrial membrane potential, and DNA damage. The transcriptomic profiles of apoptosis and autophagy-related genes were assessed by qPCR arrays. Bioinformatics analysis allowed estimation of the main gene interaction networks. Pre-screening showed that GMSM-K and Cal-27 cells were non-responsive or exhibited non-specific toxicity at high doses; therefore, subsequent analyses focused on Ca9-22 (GC) and SCC-9 (TC). HCQ significantly reduced viability and colony formation in Ca9-22 cells while moderately affecting SCC-9 cells. Autophagy inhibition was accompanied by compensatory up-regulation of autophagy-related genes, consistent with feedback activation of TFEB and FOXO3a pathways. Gene expression profiling and flow-cytometry analyses revealed cell-type-specific differences in apoptosis, mitochondrial potential, and DNA damage, suggesting HCQ’s selective anti-tumor potential in gingival carcinoma. These findings highlight HCQ as a repurposed adjuvant therapy that modulates autophagy and apoptosis to enhance chemosensitivity in oral cancer.

## 1. Introduction

Oral cancer (OC) is the 16th most common cancer worldwide, with a higher prevalence in men than in women [1]. It also ranks as the 15th leading cause of cancer-related mortality [2]. The incidence and mortality rates of OC tend to be higher in countries with low or medium human development index, where rapid socio-economic growth has contributed to the adoption of unhealthy lifestyles and increased exposure to environmental risk factors [3]. The development and progression of OC are strongly linked to multiple risk factors, including tobacco use (both smoking and chewing), alcohol consumption, poor oral hygiene, nitrosamine-rich diets, human papillomavirus infection, and genetic predisposition [3,4,5]. Oral squamous cell carcinoma (OSCC) remains a major health burden, accounting for over 90% of oral malignancies worldwide [6,7,8]. Current therapies combine surgery, radiotherapy, and chemotherapy; however, treatment outcomes are often limited by resistance mechanisms and toxicity. Four canonical mechanisms of drug resistance have been reported: (i) reduced intracellular drug concentration, (ii) enhanced DNA-repair capacity, (iii) increased tumor survival and dissemination pathways, and (iv) drug inactivation [9]. Consequently, there is growing interest in repurposing clinically approved agents with established safety profiles to target autophagy and apoptosis pathways in oral cancer.

The management of OC typically involves a combination of surgery, external beam radiation therapy, brachytherapy, and systemic treatments such as chemotherapy, immunotherapy, and targeted therapy [10,11,12]. Treatment decisions are guided by several factors, including tumor stage, tumor location, presence of metastasis, patient age, medical history, and both functional and cosmetic considerations. Recently, targeted therapies against the epidermal growth factor receptor, such as cetuximab, have demonstrated potential in improving disease outcomes [13,14,15]. However, chemotherapy remains the first-line treatment for OC, either as a primary option when surgery is not feasible or as an adjuvant with concurrent radiotherapy. Cisplatin alone or in combination with agents such as 5-fluorouracil (5-FU) or carboplatin continues to be the preferred regimen for treating advanced OC [16,17]. Chemotherapy exerts its anti-tumor effects through various mechanisms, including the induction of DNA (Deoxyribonucleic acid) damage, inhibition of DNA repair, and activation of tumor suppressor genes, such as p53, leading to cell cycle arrest and apoptosis [18,19]. Despite the availability of several treatment strategies, OC remains a major clinical challenge due to its high recurrence risk and poor prognosis. Although early detection can significantly improve survival rates, reaching up to 90%, the overall 5-year survival rate has yet to surpass 50% to 60% [20,21]. One of the key contributors to limited survival is the development of resistance to chemotherapy agents, particularly cisplatin and 5-FU (5-Fluorouracil) [22]. Chloroquine (CQ) was first synthesized by Andersag in 1934, then it was introduced into clinical trials in 1947 as a treatment for malaria. In 1946, Surrey et al. developed hydroxychloroquine (HCQ), a derivative of CQ that incorporates a hydroxyl group into one of its N-ethyl chains, thereby reducing its toxicity and improving its solubility [23]. CQ and HCQ, beyond their antimalarial activity, are lysosomotropic agents that impair autophagosome–lysosome fusion and modulate tumor cell survival. They are currently being repositioned in oncology trials for their capacity to enhance the efficacy of standard chemotherapeutics.

Both compounds have been used as anti-inflammatory agents and have demonstrated clinical efficacy in controlling autoimmune disorders such as systemic lupus erythematosus and rheumatoid arthritis, as well as in the treatment of infectious diseases, including HIV (human immunodeficiency virus) [24,25,26,27,28,29]. During COVID-19 (Coronavirus disease) pandemic, CQ has sparked a huge scientific debate about its use in SARS-CoV-2 (Severe acute respiratory syndrome coronavirus 2) treatment.

Beyond their immunomodulatory roles, CQ and HCQ have emerged as promising candidates for cancer therapy [30,31,32,33]. This is based on their role as autophagy inhibitors. Autophagy, a cellular recycling process often referred to as “self-eating”, plays a crucial role in overcoming cancer resistance. Notably, modulation of the autophagy pathway has been demonstrated to restore sensitivity in resistant cancer cells and significantly improve chemo/radiotherapy efficacy across a range of solid tumors [34,35,36,37,38]. Being the only FDA (Food and Drug Administration)-approved autophagy inhibitors currently available for clinical use, CQ and HCQ are being evaluated in over 30 ongoing clinical trials worldwide (http://clinicaltrials.gov/) (accessed on 3 May 2024), with encouraging preliminary outcomes. Importantly, recent research suggests that the anti-cancer properties of CQ and HCQ extend beyond autophagy inhibition. These agents have been shown to induce lysosomal membrane permeabilization, triggering apoptosis independently of autophagy blockade [39]. This mechanism has been observed in various cancer models, including lung and breast cancers, where CQ enhanced sensitivity to PI3K/mTOR inhibitors [32,40,41]. Previous studies have demonstrated the ability of HCQ to inhibit autophagy and increase cell death in several solid tumors. However, its specific impact on oral epithelial carcinomas and its relationship with tumor-type-specific responses remain poorly defined. Here we compare the effects of CQ and HCQ on tongue (SCC-9—TC) and gingival (Ca9-22—GC) carcinoma cells, representing two clinically distinct OSCC subtypes, to identify differential autophagy and apoptosis responses. This study aims to (1) evaluate the selective cytotoxicity of CQ and HCQ toward oral carcinoma cell lines, (2) analyze their effects on autophagy, apoptosis, and DNA damage, and (3) explore gene-expression profiles to understand cell-type-specific mechanisms.

## 2. Results

### 2.1. CQ Presents High Efficacy Against Tongue Carcinoma, While HCQ Is More Selective for Gingival Carcinoma

**Figure 1:**
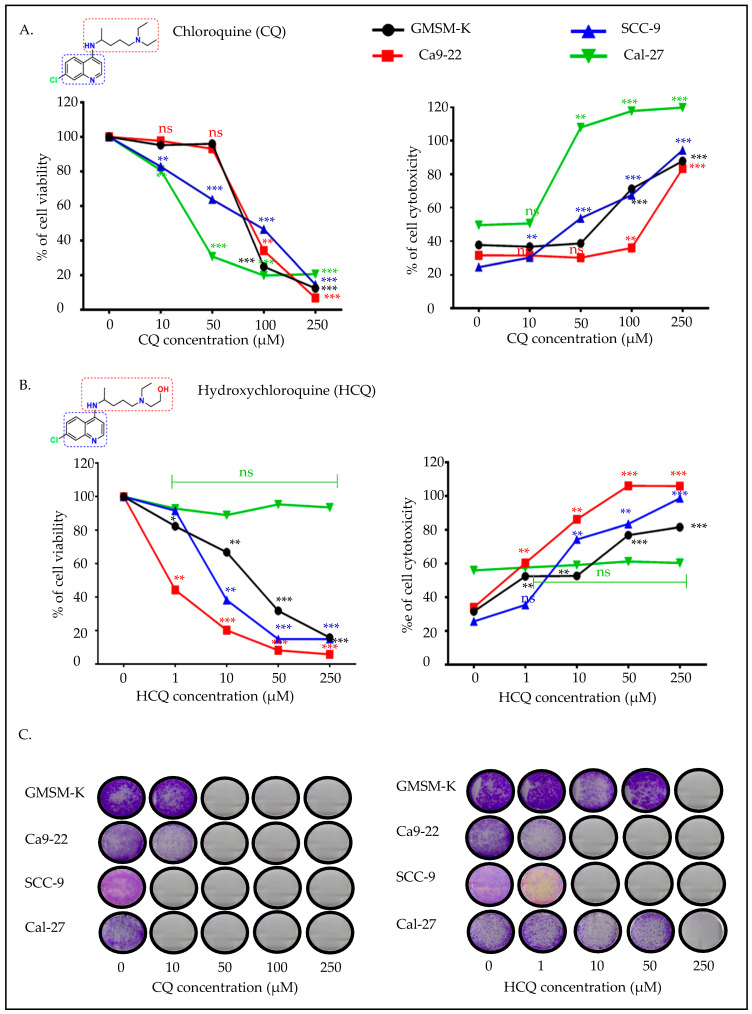
Effect of CQ and HCQ on cell viability, cytotoxicity and colony formation. GMSM-K (*n = 12*), Ca9-22 (*n = 6*), SCC-9 (*n = 6*), and Cal-27 (*n = 6*) were treated with different concentrations of CQ (0, 10, 50, 100, and 250 μM) or HCQ (0, 1, 10, 50, and 250 μM) for 24 h. MTT and LDH assays were performed to evaluate cell viability and cytotoxicity following exposure to (**A**) CQ or (**B**) HCQ. (**C**) Crystal violet staining was performed after 14 days of exposure to both treatment regimens to evaluate colony formation capacity (*n = 6*). Comparisons are presented relative to untreated controls. All data are expressed as mean ± SEM. * *p* < 0.05; ** *p* < 0.01; *** *p* < 0.001 were considered statistically significant; ns: non-significant.

Cell viability, cytotoxicity, and colony formation were evaluated in different OC cell lines as compared to the GMSM-K non-oncogenic cell line treated with increasing concentrations of CQ or HCQ. As shown in Figure 1, Ca9-22 (GC) and SCC-9 (TC) cells showed dose-dependent reductions in viability after CQ and HCQ exposure, whereas GMSM-K and Cal-27 cells remained resistant. Cal-27 displayed non-specific toxicity only at high doses (≥100 µM) and was excluded from subsequent analyses. These results were further confirmed by the LDH (lactate dehydrogenase) assay, where CQ induced a strong cytotoxicity at low doses in tongue cancer cells (Figure 1A). On the other hand, Ca9-22 gingival cancer cells showed the highest sensitivity to HCQ, with a significant impact observed at 1 µM. At this concentration, HCQ reduced Ca9-22 cell proliferation by approximately 50%, without significantly affecting normal epithelial cells or tongue cancer cells. At concentrations above 10 µM, the inhibitory effect became even more pronounced in Ca9-22 cells. HCQ also appeared to be effective against SCC-9 tongue carcinoma cells, with no effect observed in Cal-27 cells. These results were confirmed by the LDH assay (Figure 1B). Prolonged treatment for over two weeks with CQ inhibited new colony formation mainly in tongue cancer cells (Cal-27 and SCC-9). A minor effect was recorded in the presence of Ca9-22 cells, and this is relative to GMSM-K. Concentrations measuring 50 µM and above appeared to be non-cancer specific. On the contrary, HCQ treatment mainly impacted Ca9-22, with SCC-9 being affected to a lesser extent. No effect on GMSM-K or Cal-27 colony formation was recorded at 50 µM concentration and below (Figure 1C). Given the limited response of Cal-27 to HCQ, the rest of this study is focused on comparing the effect of CQ and HCQ on the Ca9-22 and SCC-9 cell lines.

### 2.2. CQ and HCQ Inhibit Autophagy, with Gingival Carcinoma Being More Sensitive to HCQ than Tongue Carcinoma

**Figure 2:**
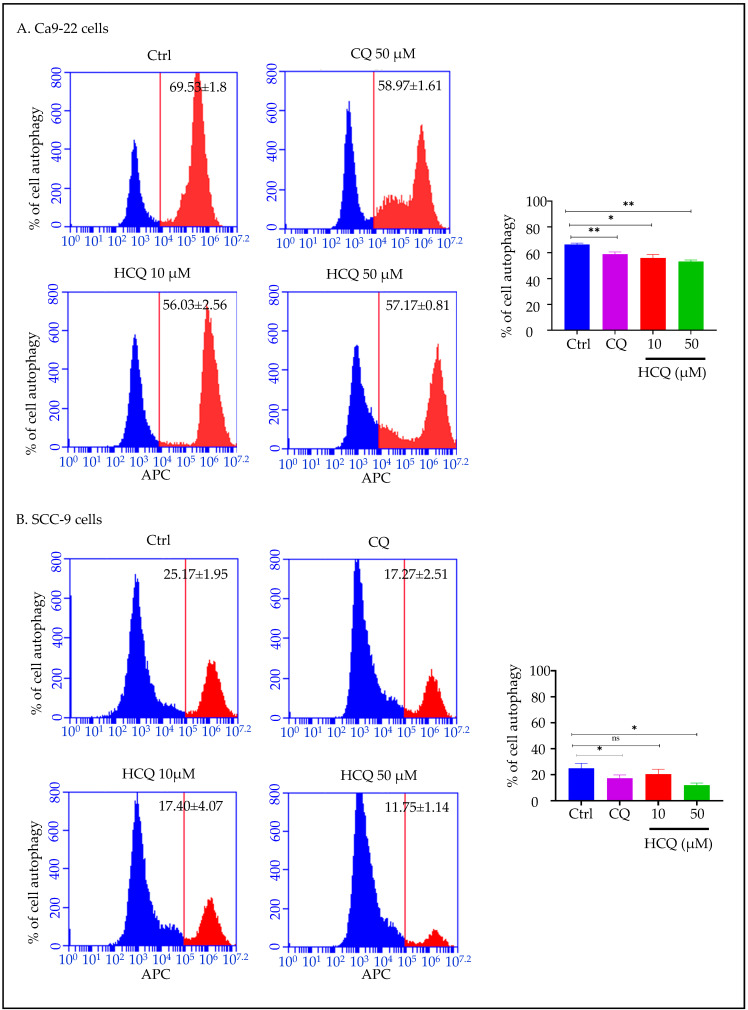
Effect of CQ and HCQ on autophagy inhibition. (**A**) Ca9-22 and (**B**) SCC-9 were treated either with 50 µM CQ or with HCQ at 10 and 50 µM, and this is for 24 h (*n = 3*). Autophagy levels were measured by flow cytometry using a red fluorescent label targeting autophagosomes and autolysosomes. Results are expressed as mean ± SEM. * *p* < 0.05; ** *p* < 0.01 were considered statistically significant; ns: non-significant.

To assess whether the anti-cancer effect of CQ and HCQ on the OC cells is associated with autophagy inhibition, we measured the percentage of autophagic cells by flow cytometry. Both CQ (50 µM) and HCQ (10 and 50 µM) effectively reduced autophagy in Ca9-22 and SCC-9 OC cells. More specifically, the percentage of autophagic cells decreased from 69.53 ± 1.8% in untreated cells to 58.97 ± 1.61% with 50 µM CQ, and 57.17 ± 0.81% with 50 µM HCQ (Figure 2A). In SCC-9 cells, a similar effect was observed. The autophagic population significantly decreased from 25.17 ± 1.95% at baseline to 17.27 ± 2.51% and 11.75 ± 1.14% with 50 µM CQ and 50 µM HCQ (Figure 2B). HCQ at low dose measuring 10 µM induced a more pronounced reduction in autophagy in Ca9-22 cells (*p* < 0.005) as compared to SCC-9, where no significant effect was registered, thus highlighting its strong potential to modulate autophagy in gingival squamous cell carcinoma.

### 2.3. CQ and HCQ Caused Moderate Accumulation of Autophagosomes, Accompanied by Up-Regulation of Autophagy-Related Genes, Indicating Compensatory Feedback Activation via TFEB

**Figure 3:**
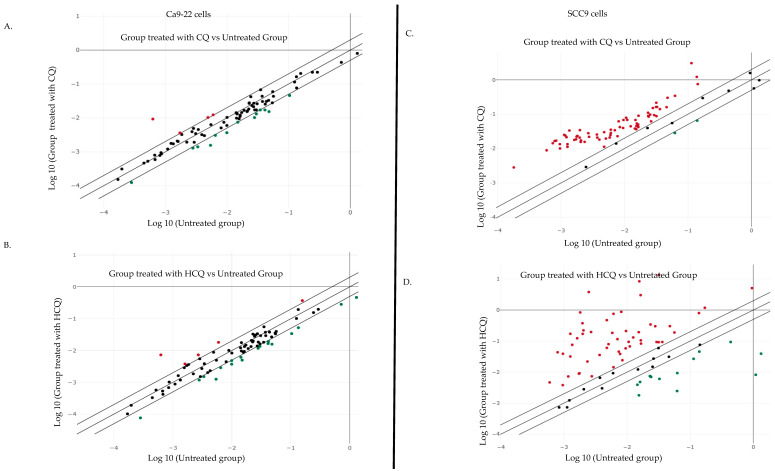
Correlation scatter plots of autophagy-related gene expression in (**A**) Ca9-22 treated with 10 µM of CQ and (**B**) Ca9-22 treated with 50 µM of HCQ. (**C**) SCC-9 cells under CQ treatment. (**D**) SCC-9 cells under HCQ treatment (*n = 3*).

To further explore the differences in CQ and HCQ mechanisms of action on Ca9-22 and SCC-9 OC cells, QPCR (quantitative polymerase chain reaction) arrays screening for autophagy gene expression were performed. As shown in Figure 3, different pathways were modulated in gingival and tongue carcinoma cell lines in response to both drugs. For instance, exposure to 50 µM CQ modulated the expression of 17 out of 84 autophagy-related genes in Ca9-22 cells. Among these, four genes were significantly upregulated (≥2-fold), and 13 genes were downregulated. HCQ at 10 µM, on the other hand, altered the expression of 23 genes, with 5 upregulated and 18 downregulated (Figure 3A and Supplementary Table S1). In SCC-9 cells, both CQ at 50 µM and HCQ at 10 µM modulated a greater number of autophagy-related genes as compared to Ca9-22 cells. More specifically, CQ treatment modulated 72 out of the 84 autophagy-associated genes analyzed. Among these, 70 genes were significantly upregulated, with changes ranging from 2.36-fold (e.g., *LAMP1*, *NFKB1*) to 27.02-fold for *MAP1LC3B*. Similarly, although HCQ at 10 µM did not show any significant effect on autophagy at 24 h, the drug modulated the expression of 65 autophagy-related genes, suggesting a possible delayed impact on the autophagic process. Among the 53 upregulated genes were several members of the *ATG* gene family, which play a central role in autophagosome formation and lysosomal degradation. These included *ATG4D* and *ATG9B*, whose expression levels increased by 136.24-fold and 40-fold, respectively. Among the 12 downregulated genes, *BNIP3* (−33.82-fold) and *HSPA8* (−133.44-fold) were the most significantly affected (Figure 3B and Table S2).

**Figure 4:**
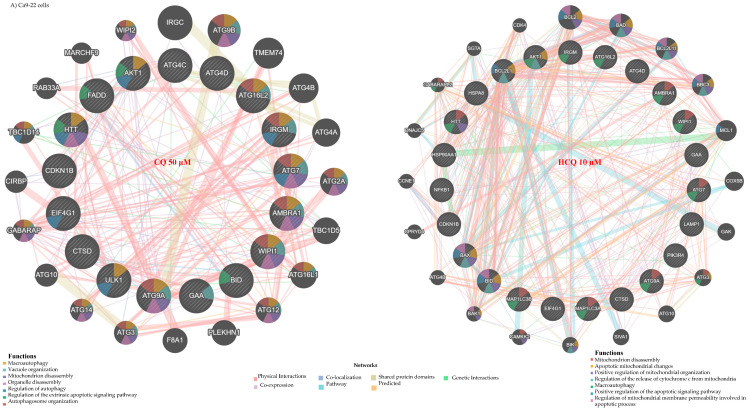

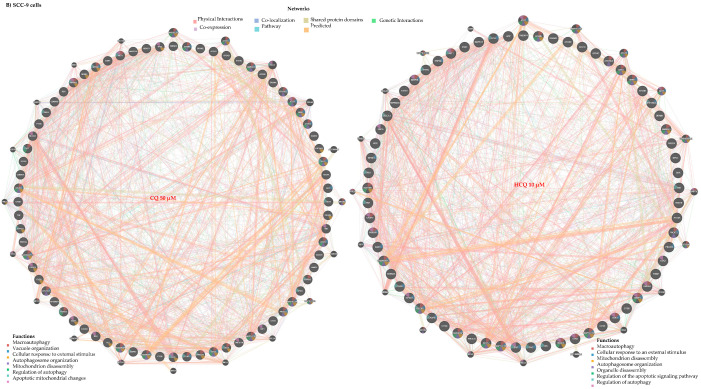
Gene expression profiles and interaction networks of autophagy-related markers. Genes modulated by CQ (50 µM) and HCQ (10 µM) in (**A**) Ca9-22 (*n = 3*) and (**B**) SCC-9 (*n = 3*) are presented. Only genes showing at least a two-fold variation relative to controls were considered. Interaction networks were generated using Gene MANIA (genemania.org) to illustrate possible gene connections and key biological functions affected by each treatment.

The autophagy genes modulated by CQ and HCQ in both types of OC cells are predicted to form distinct interaction networks. The Gene MANIA (Version 3.5.3, released on 29 February 2024) tool estimated that specific sets of genes can be highly interconnected through co-expression, physical interactions, and shared protein domains, among other possible interaction options. In Ca9-22 gingival cells, analysis of the genetic interaction network revealed that the genes modulated by CQ are possibly linked to genes involved in key biological functions such as autophagosome organization, vacuole organization, and organelle disassembly. Moreover, connections to mitochondrion disassembly and the extrinsic pathway of apoptosis are anticipated, suggesting a blockage of autophagy and a direction towards programmed cell death. In contrast, the interaction network of genes modulated by HCQ is predominantly expected to be associated with mitochondria-related biological processes. Functional analysis revealed potential involvement in mitochondrial disassembly, regulation of mitochondrial membrane permeability, and activation of the intrinsic (mitochondria-dependent) apoptotic pathway. Key genes contributing to these processes include *BCL2*, *BAD*, *BID*, *BAX*, and *BBC3* (Figure 4A). In SCC-9 cells, the network is even more complex due to the large number of genes modulated by CQ and HCQ. These genes are capable of interacting with other regulators involved in functions, such as autophagy modulation, macro-autophagy, autophagosome organization, and mitochondrial apoptotic pathways (Figure 4B).

### 2.4. CQ and HCQ Reduce Mitochondrial Membrane Potential, with Gingival Carcinoma Being More Sensitive to HCQ than Tongue Carcinoma

**Figure 5:**
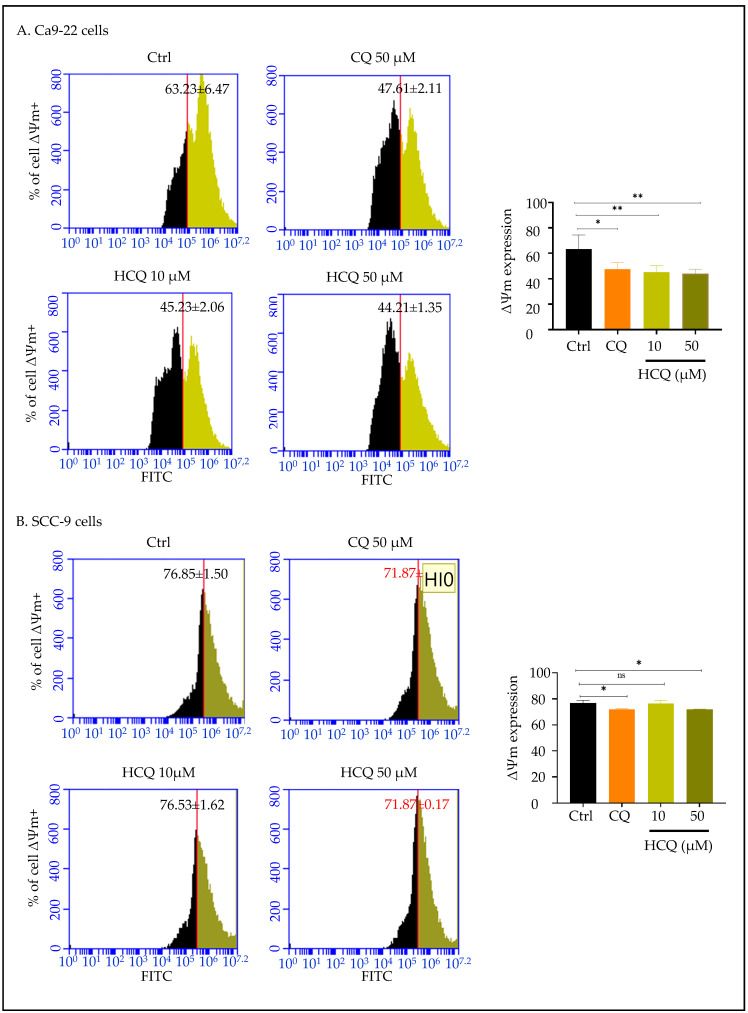
Effect of CQ and HCQ on Mitochondrial Membrane Potential (Δψm). (**A**) Ca9-22 (*n = 4*) and (**B**) SCC-9 (*n = 3*) were treated with CQ and HCQ for 24 h. Flow cytometry analysis of mitochondrial membrane potential was performed using the DiOC_2_ marker. Results are expressed as mean ± SEM. * *p* < 0.05; ** *p* < 0.01 were considered statistically significant; ns: non-significant.

To confirm mitochondrial dysfunction, we next assessed the change in the mitochondrial membrane potential (Δψm) in Ca9-22 and SCC-9 cells following treatment with CQ and HCQ. As shown in Figure 4, CQ and HCQ led to a significant reduction in Δψm in both OC cell types. More in detail, the proportion of cells with high membrane potential decreased from 63.23 ± 6.47% in Ca9-22 control cells to 45.23 ± 2.06% and 44.21 ± 1.35% in cells treated with 10 µM and 50 µM HCQ. A similar effect was also observed with CQ at 50 µM, with a reduction reaching 15.62% (Figure 5A). In contrast, SCC-9 cells showed no significant change in Δψm with HCQ at 10 µM. This finding indicates that HCQ induces mitochondrial depolarization more selectively in Ca9-22 cells as compared to SCC-9 cells. Moreover, the proportion of tongue cells with high membrane potential changed from 76.85 ± 1.50% in control to 71.87 ± 0.17% with HCQ and CQ at 50 µM (Figure 5B).

### 2.5. CQ and HCQ Modulate GSH Levels Differently Across Tongue and Gingival Cell Lines

**Figure 6:**
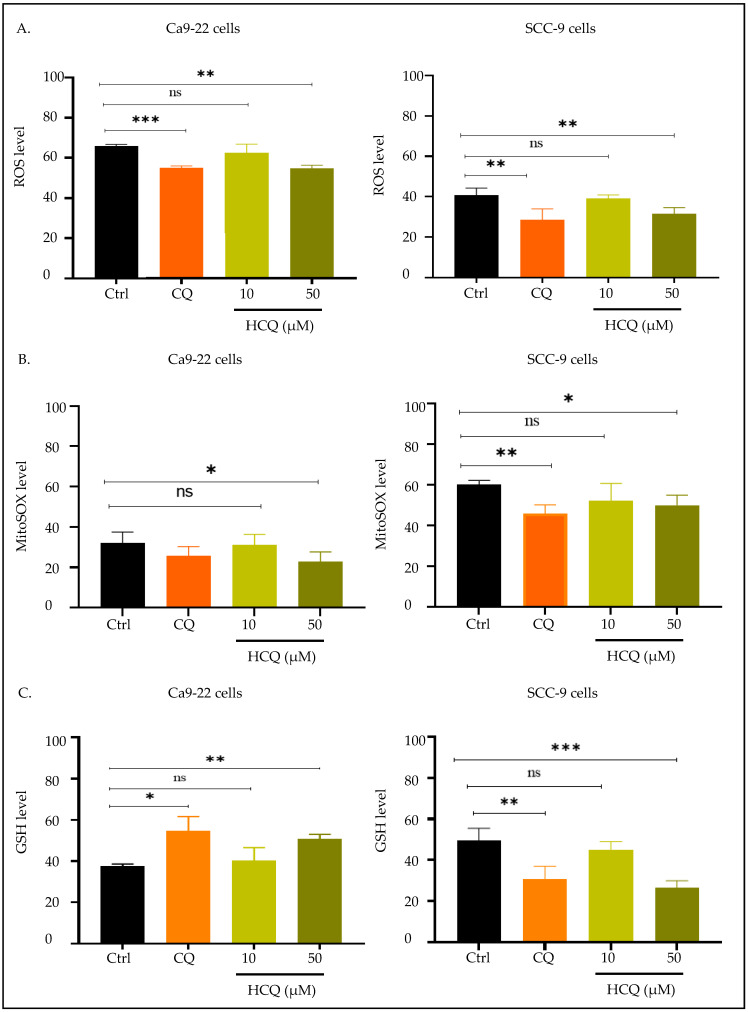
Effect of CQ and HCQ on oxidative stress. Comparative analysis of CQ and HCQ’s effects on oxidative stress in Ca9-22 and SCC-9 cells after 24 h treatment (*n = 4*). Drugs’ effects on (**A**) total ROS, (**B**) mitochondrial ROS, and (**C**) GSH levels were assessed by flow cytometry. Results are expressed as mean ± SEM. * *p* < 0.05; ** *p* < 0.01; *** *p* < 0.001 were considered statistically significant; ns: non-significant.

To understand the mechanism by which CQ and HCQ exert their anti-cancer effects against gingival and tongue squamous carcinoma cells, we evaluated their effect on total and mitochondrial oxidative stress, as well as on GSH antioxidant levels. Our results showed that both CQ and HCQ treatments at 50 µM reduced total ROS and MitoSOX levels in Ca9-22 and SCC-9 cell lines (Figure 6A,B). In contrast, GSH levels increased in gingival carcinoma cells under the influence of CQ and HCQ. To be precise, the intracellular levels of GSH augmented from 37.65 ± 0.7% in Ca9-22 control cells to 54.70 ± 4.93% and 50.88 ± 1.52% in the cells treated with CQ and HCQ at 50 µM. Surprisingly, inverse results were observed in SCC-9 cells, where a significant decrease in GSH levels was observed after exposure to CQ and HCQ at the same concentration. GSH values decreased from 49.45 ± 3.0% to 30.85 ± 3.04% with CQ, and to 26.52 ± 1.69% with HCQ treatment (Figure 6C).

### 2.6. HCQ Triggers DNA Damage in Gingival Cells, with No Detectable Effect in Tongue Carcinoma

**Figure 7:**
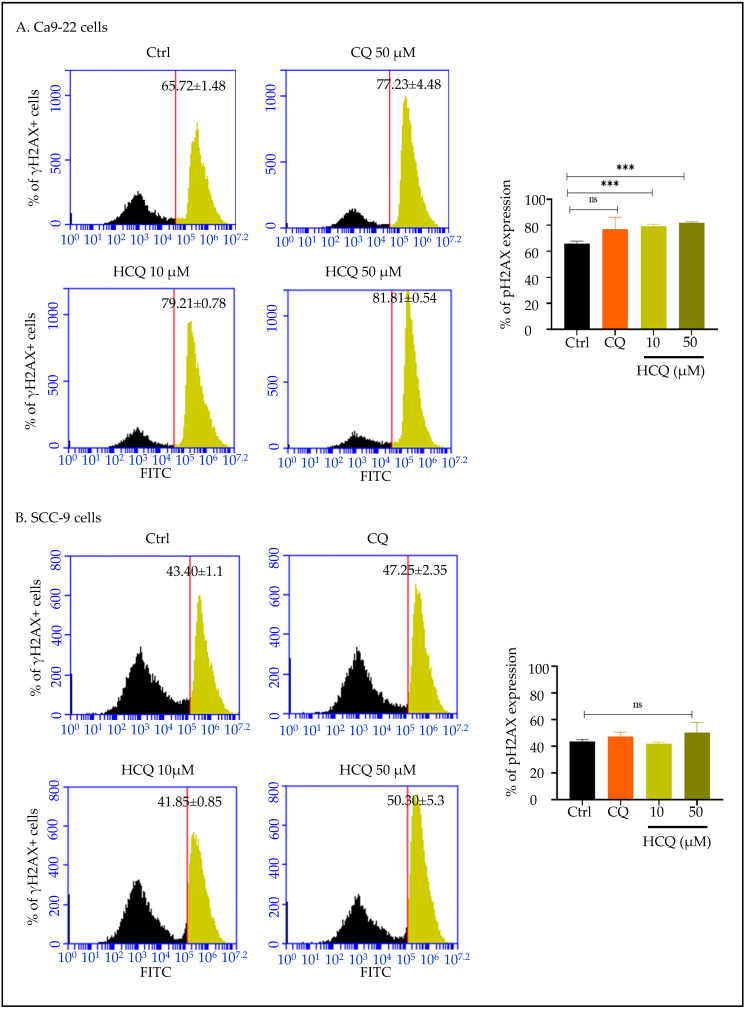
Effect of CQ and HCQ on DNA damage. (**A**) Ca9-22 and (**B**) SCC-9 were exposed to CQ and HCQ for 24 h (*n = 3*). Flow cytometry analysis allowed detection of phosphorylated H2AX levels. Results are expressed as mean ± SEM. *** *p* < 0.001 was considered statistically significant; ns: non-significant.

DNA damage was evaluated using flow cytometry. As shown in Figure 6**,** treatment with HCQ at 10 and 50 µM significantly increased the phosphorylation of histone H2AX (γH2AX) in Ca9-22 cells, indicating the presence of DNA double-strand breaks. Overall, the percentage of γH2AX-positive cells increased from 65.72 ± 1.48% in the controls to 79.21 ± 0.78% and 81.81 ± 0.54% following exposure to HCQ treatment at 10 µM and 50 µM, respectively. Importantly, CQ at 50 µM was not effective at this level (Figure 7A).

In SCC-9 cells, no significant impact was observed in the presence of CQ and HCQ (Figure 7B).

### 2.7. HCQ Selectively Induces Apoptosis in Human Gingival Carcinoma Cells

**Figure 8:**
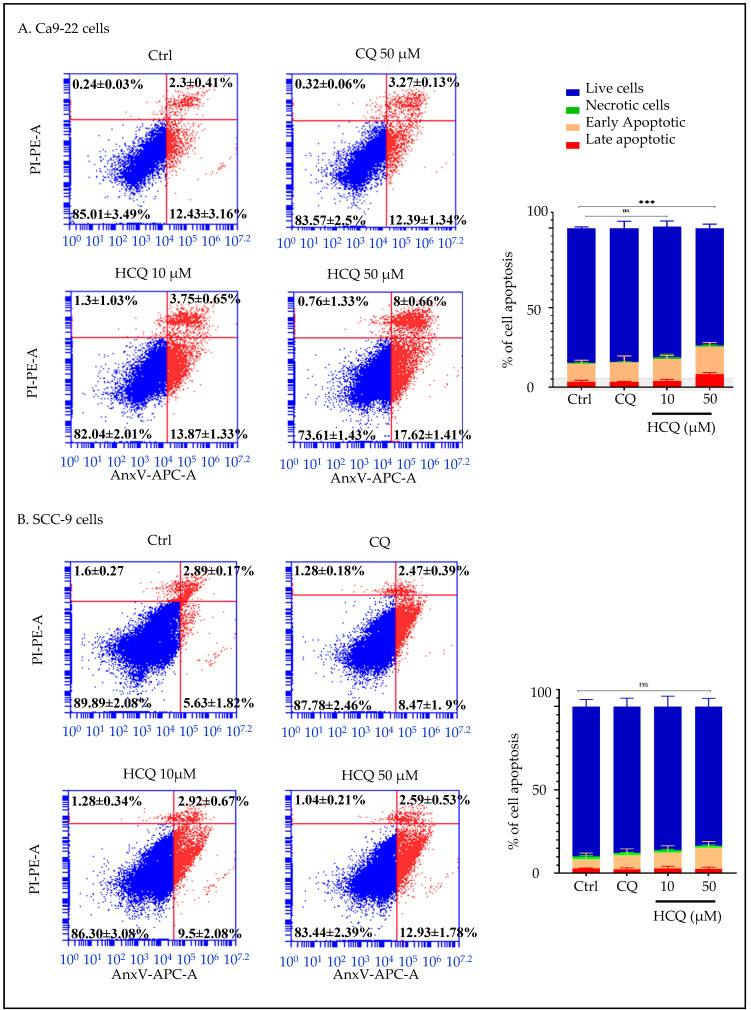
Effect of CQ and HCQ on apoptosis induction. (**A**) Ca9-22 (*n = 3*) and (**B**) SCC-9 (*n = 4*) were treated with CQ and HCQ for 24 h. Flow cytometry analysis classified cells into viable, early apoptotic, late apoptotic populations based on AnxV and PI staining. The percentages of cells in each phase are expressed as mean ± SEM. *** *p* < 0.0005 indicates statistical significance; ns: not significant.

Flow cytometry analysis using the AnxV/PI kit revealed that HCQ at the highest concentration, measuring 50 µM, specifically enhances apoptosis in gingival carcinoma cells. More specifically, in Ca9-22, the percentage of dead cell populations increased from 14.99% in untreated controls to 26.39% in cells incubated with 50 µM HCQ (*p* < 0.0005). Importantly, CQ did not affect the Ca9-22 apoptotic process (Figure 8A). Additionally, both CQ and HCQ showed no effect on SCC-9 cell death, with apoptotic rates fluctuating from 10.11% in controls to 12.22% and 16.56% in the presence of CQ and HCQ at 50 µM (Figure 8B). These results indicate one more time that Ca9-22 cells are more susceptible than SCC-9 cells to HCQ-induced mortality, specifically via apoptosis.

### 2.8. HCQ and CQ Differentially Modulate the Expression of Apoptosis-Related Genes Depending on the Type of OC Cells

**Figure 9:**
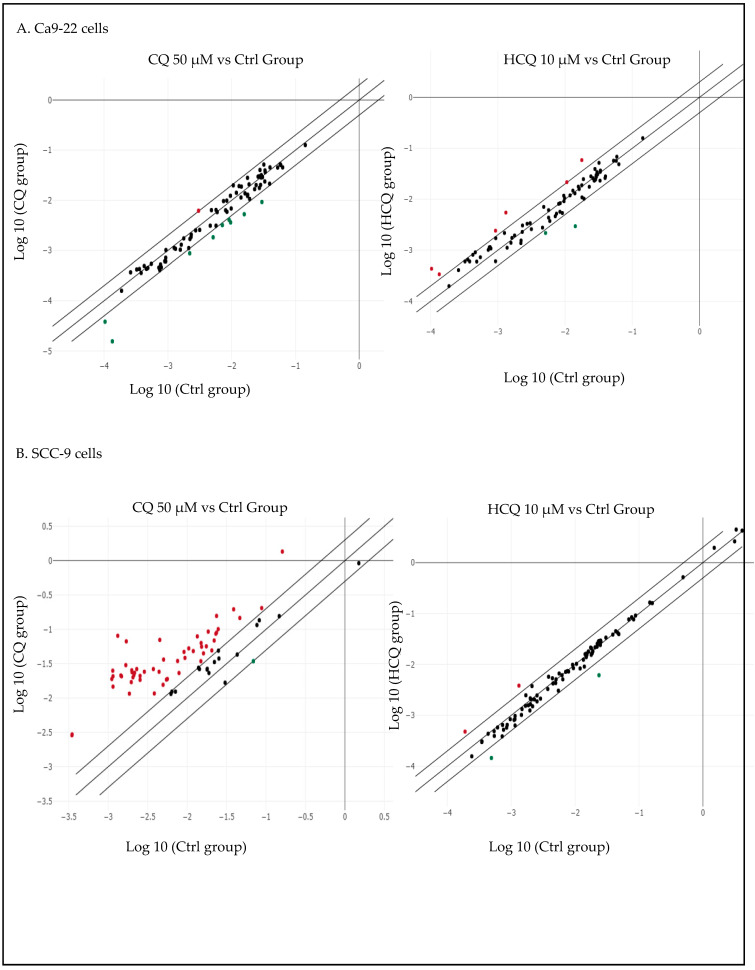
Correlation scatter plots of apoptosis-related gene expression in (**A**) Ca9-22 and (**B**) SCC-9 under CQ and HCQ treatment (*n = 3*).

In gingival carcinoma cells, CQ treatment at 50 µM up-regulated 6 out of 84 apoptosis-related genes by at least twofold as compared to untreated cells. These genes include *BAK1* (2.04-fold), *BCL2A1* (2.61-fold), *CYCS* (4.16-fold), *DAPK1* (4.22-fold), *RIPK2* (3.31-fold), and *TNFRSF11* (2.52-fold). Conversely, two genes, *CASP2* and *XIAP*, were downregulated by 2.35-fold and 4.80-fold, respectively. On the other side, treatment with 10 µM HCQ induced the expression of only one pro-apoptotic gene (*BIK*/2.05-fold) while reducing the expression of 9/84 apoptotic markers, namely: *APAF1* (−2.23-fold), *BIRC2* (−3.22-fold), *BIRC5* (−2.65-fold), *CASP2* (−2.80-fold), *CASP6* (−2.98-fold), *DAPK1* (−2.71-fold), *TNFRSF11* (−8.67-fold), *TRADD* (−2.51-fold), and *TRAF2* (−2.23-fold) (Figure 9A and Table S3). In SCC9 cells, 50 µM CQ treatment upregulated the expression of 53 apoptotic genes, and only one gene (*TNFSF10*) was downregulated. Notably, *CYCS* was regulated by more than 61-fold, while *CIDEA* expression levels increased by 21.89-fold. Finally, 10 µM HCQ treatment induced 2 apoptotic genes (*CYCS* and *DAPK1*) while downregulating anti-apoptotic *BCL2* and *BIRC6* (Figure 9B and Table S4).

**Figure 10:**
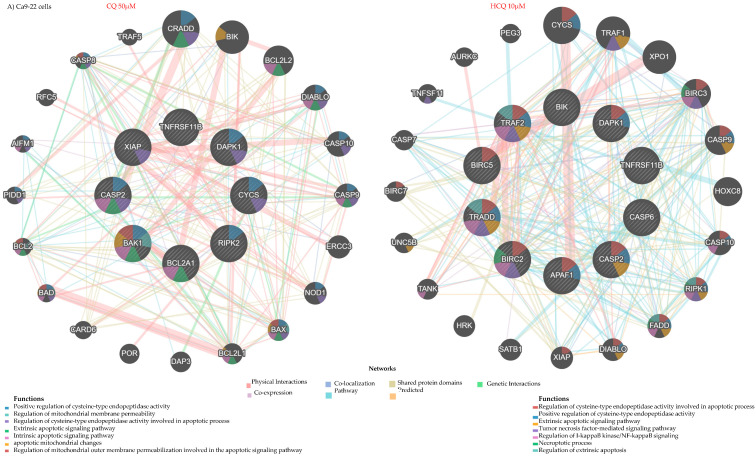

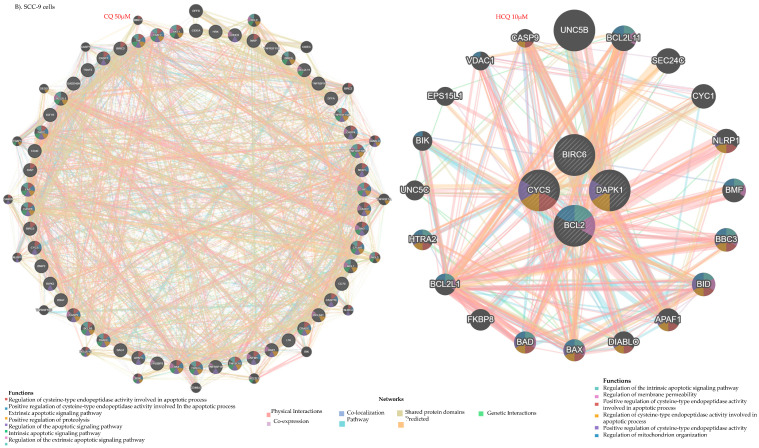
Gene expression profiles and interaction networks of apoptosis-related markers. Genes modulated by CQ (50 µM) and HCQ (10 µM) in (**A**) Ca9-22 (*n = 1*) and (**B**) SCC-9 (*n = 1*) are presented. Only genes showing at least a two-fold variation relative to controls were considered. Interaction networks were generated using Gene MANIA (genemania.org) to illustrate possible gene connections and key biological functions affected by each treatment.

Based on the distinct gene expression profiles observed across different cell lines and treatment types, the analysis of apoptotic gene interaction networks suggests involvement in various biological functions. For instance, in Ca9-22 cells, the genes modulated by CQ and HCQ are organized into functional networks centered around mitochondrial apoptosis. These genes can be notably involved in regulating mitochondrial membrane permeability, a key event in the activation of the intrinsic apoptotic pathway. Functional analysis also highlights their role in the regulation of cysteine-type endopeptidase activity, such as caspases, and in the release of cytochrome c, which initiates the apoptotic cascade (Figure 10A). In SCC-9 cells, CQ modulated a larger number of apoptotic genes than HCQ, resulting in a more complex interaction network. This network includes genes involved in the extrinsic apoptotic pathway (TNFSF10, TRADD), in addition to the intrinsic pathway, suggesting an activation of cell death. Although HCQ modulates only 4 genes in SCC-9 cells, its repression of anti-apoptotic genes such as BCL2 and BIRC6 may enhance cellular sensitivity to apoptosis (Figure 10B).

## 3. Discussion

Platinum-based chemotherapeutic drugs, including cisplatin, paclitaxel, and fluorouracil, remain the standard of care for OC. Their long-term efficacy is limited by their high toxicity and the development of drug resistance. Autophagy has emerged as a key mechanism contributing to chemoresistance in various cancers, including OC, making the inhibition of this process an attractive therapeutic strategy [34,42,43]. CQ and HCQ are currently the only clinically approved autophagy inhibitors. HCQ, a hydroxylated derivative of CQ, offers better solubility and a more favorable safety profile [44,45,46], which has increased clinical interest in its therapeutic application. It is now being used either as monotherapy or in combination with radiotherapy or chemotherapy for the treatment of patients with solid and hematological tumors [47,48]. A meta-analysis of seven trials evaluating the addition of CQ or HCQ to standard cancer therapy across different types of cancer (glioblastoma, brain metastases from non-small cell lung cancer and breast cancer, non-Hodgkin lymphoma, and pancreatic adenocarcinoma) concluded that their use was associated with improvements in overall response rate, progression-free survival, and overall survival [49]. Although many studies have reported that antitumor effects of CQ via autophagy inhibition in cancers such as breast cancer [50], melanoma [31,51], pancreatic [52], lung, and colorectal cancers [53], data on its use in OC, particularly gingival carcinoma, remain limited. In this context, our study is the first to compare the anti-tumor effects of CQ and HCQ in both gingival and tongue squamous carcinoma cell lines.

This study demonstrates that HCQ exerts a selective cytotoxic effect on gingival carcinoma (Ca9-22) compared to tongue carcinoma (SCC-9) cells. The moderate inhibition of autophagy, coupled with gene up-regulation, suggests blockade of autophagic flux and compensatory activation of TFEB-mediated transcription. Notably, HCQ was effective at low concentrations (1μM), highlighting its therapeutic potential. Mechanistically, while both drugs reduced autophagy in tongue and gingival cancer cells, our transcriptomic analysis indicated that HCQ modulated a different array of autophagy-related genes compared to CQ, reflecting divergent molecular mechanisms of action. In Ca9-22 cells, HCQ downregulated key autophagy genes such as *ATG7*, *ATG9A*, *AMBRA1*, and *LAMP1*, which are essential for autophagosome formation and lysosomal degradation, whereas CQ primarily affected genes involved in canonical autophagy pathways. These results align with previous studies, showing that CQ and HCQ inhibit autophagy predominantly by preventing autophagosome-lysosome fusion rather than solely by elevating lysosomal pH [54,55]. Additionally, both drugs have been reported to cause autophagy-independent disruption of the Golgi and endo-lysosomal systems, potentially contributing to impaired cellular homeostasis [55,56,57]. Furthermore, gene interaction network analysis revealed that HCQ preferentially targeted mitochondrial-related pathways, including those involved in the regulation of mitochondrial membrane permeability and intrinsic apoptosis signaling.

Consistent with this, we found that both CQ and HCQ likely promote mitochondrial membrane permeabilization in gingival and tongue cancer cells, with gingival carcinoma showing greater sensitivity to HCQ. Cancer cells often exhibit higher ΔΨm levels compared to normal cells, a feature linked to increased tumor invasive properties in vitro and enhanced metastases in vivo. This elevated ΔΨm in cancer cells results from various factors, including changes in ATP synthase activity and altered ion transport [58,59]. In line with our findings, CQ was found to reduce oxidative phosphorylation and electron transport chain activity in adipocytes, while generating metabolic dysregulation [60]. Similarly, studies suggest that HCQ, like its parent compound CQ, can alter ΔΨm and cause mitochondrial dysfunction. Notably, a study by Patricia Boya and colleagues has shown that HCQ induces mitochondrial membrane permeabilization, BAX activation, and cytochrome c release, supporting our hypothesis that HCQ engages mitochondrial pathways to promote apoptosis [61].

Targeting the redox system in cancer involves modulating the balance between ROS and antioxidants to induce cancer cell death. Cancer cells often display a disrupted redox balance. Importantly, ROS play a dual role in cancer biology: at moderate levels, they stimulate proliferation, angiogenesis, and metastasis by activating factors such as VEGF and HIF-1α, whereas excessive ROS levels can trigger cytotoxicity [62,63]. In our study, both CQ and HCQ significantly reduced intracellular ROS and mitochondrial superoxide levels in Ca9-22 and SCC-9 cells. This observed reduction in ROS contrasts with the mechanism of many chemotherapeutic drugs, which often rely on ROS elevation to trigger oxidative stress-mediated apoptosis [64]. Supporting our findings, a study on curcumin-treated breast cancer cells reported that curcumin-induced ΔΨm depolarization was accompanied by a reduction in ROS levels [65]. Another study found that curcumin suppressed proliferation, migration, and invasion of pancreatic cancer cells by decreasing ROS generation and inhibiting the ERK/NF-κB signaling pathway [66,67]. While both drugs increased GSH levels in Ca9-22 cells, they reduced GSH in SCC-9 cells, suggesting tumor origin-specific redox responses. Our findings are consistent with those of Alexandre Teixeira Vessoni and colleagues, who observed that CQ-induced glioma cell death was associated with a loss of mitochondrial membrane potential, without a corresponding increase in oxidative stress [67]. Furthermore, another recent study using a murine model of tongue OSCC demonstrated that CQ, when combined with dichloroacetate (DCA) and arsenite, improved survival and reduced tumor development. Notably, while arsenite alone lowered ROS levels, the addition of CQ and DCA further enhanced oxidative balance by boosting antioxidant enzymes such as catalase and superoxide dismutase [68]. However, elevating ROS together with antioxidant defenses is not always advantageous, as certain cancer cells may exploit this balance to promote tumor progression [62,63]. Overall, our results suggest that CQ and HCQ contribute to tumor suppression by fine-tuning oxidative balance in a context-dependent manner.

In addition to its effects on autophagy, mitochondrial dysfunction, and redox balance, we found that HCQ only generated DNA damage in Ca9-22 gingival carcinoma cells, as evidenced by the increased levels of phosphorylated γH2AX. This effect was not observed with CQ treatment. γH2AX serves as a reliable marker of DNA double-strand breaks, indicating genomic instability and activation of DNA damage response pathways. To date, very limited data is available on the genotoxic potential of HCQ in cancer. The first study to report HCQ-induced DNA damage was conducted in 2022 by Ahmad Besaratinia et al., who demonstrated a significant increase in 8-oxodG levels in primary mouse embryonic fibroblasts following HCQ treatment. However, only one study has explored this effect in a cancer context. Chen et al. reported that HCQ modulates autophagy and induces DNA damage in hepatocellular carcinoma, helping to overcome sorafenib resistance via the TLR9/SOD1/hsa-miR-30a-5p/Beclin-1 axis [69]. In contrast, our findings regarding CQ do not align with previous studies that reported CQ-induced DNA damage in various cancer types [70], including tongue carcinoma cell lines. For example, CQ was shown to enhance cytotoxicity by inducing DNA damage, demonstrating significant efficacy in glioblastoma and head and neck squamous carcinoma models, such as CAL-33 tongue carcinoma cells [71]. Higher γH2AX signal and mitochondrial depolarisation in Ca9-22 cells suggest oxidative stress contributes to HCQ-induced DNA damage. This aligns with findings that 8-hydroxy-2′-deoxyguanosine (8-OHdG) marks oxidative DNA damage in OSCC [72].

Finally, we expected that the cumulative effects of HCQ on autophagy inhibition, mitochondrial dysfunction, redox regulation, and DNA damage would ultimately lead to cell death. To confirm this hypothesis, we measured apoptotic cell levels and found that HCQ, and not CQ, induced apoptosis solely in Ca9-22 gingival carcinoma cells. This finding is consistent with previous reports indicating that CQ fails to activate apoptosis in tongue squamous carcinoma models [71]. On the other hand, multiple studies have demonstrated the pro-apoptotic effects of HCQ in various cancer types. For instance, Hoque et al. showed that HCQ synergistically inhibited Bcl-xL and induced apoptosis in pancreatic cancer cells, both in vitro and in vivo [73]. Another study reported that autophagy blockade by HCQ significantly increased apoptosis in lung cancer cells by activating both the extrinsic (FOXO3a/FasL/caspase-8) and intrinsic (caspase-9) apoptotic pathways [74]. Furthermore, a clinical study demonstrated that HCQ induces the secretion of the tumor suppressor Par-4, resulting in apoptosis activation and metastasis inhibition. Notably, elevated plasma Par-4 levels correlated with apoptosis in resected tumors, further supporting HCQ’s pro-apoptotic potential in cancer [75].

Amid these observations, our transcriptomic analysis revealed differences in the apoptotic signaling pathways modulated by CQ and HCQ in Ca9-22 gingival carcinoma cells and SCC-9 tongue carcinoma cells. More specifically, in SCC-9 cells, both CQ and HCQ altered broad gene networks related to intrinsic and extrinsic apoptosis compared to Ca9-22 cells; however, these changes did not result in functional apoptosis. A similar pattern was observed in Ca9-22 cells treated with CQ, where transcriptional modulation is expected to primarily affect genes involved in mitochondrial membrane permeability and extrinsic apoptotic signaling, without corresponding apoptotic activity. The transcriptomic changes suggest that while both drugs may prime apoptotic pathways at the gene level, this does not necessarily translate into functional apoptosis detectable by the AnxV/PI assay, possibly due to post-transcriptional regulation, assay limitations, or timing of the experiment.

In contrast, HCQ modulated a broader gene network in Ca9-22 cells and successfully induced apoptosis, with the affected genes predominantly involved in the extrinsic apoptotic pathway. Several genes regulated by HCQ in Ca9-22 cells have been identified as potential cancer therapy targets, including TNFRSF11B, CASP6, BIRC2, and BIRC5. TNFRSF11B, a member of the tumor necrosis factor receptor superfamily [76], is highly expressed in several tumor types, including oral squamous cell carcinoma, and is associated with increased tumor cell survival, angiogenesis, and metastatic potential [77,78]. TNFRSF11B can protect cancer cells from apoptosis by sequestering TRAIL, thereby blocking TRAIL-induced extrinsic apoptotic signaling. Its downregulation by approximately 9-fold in Ca9-22 cells suggests efficacy of HCQ treatment through induction of apoptosis. By consulting the UALCAN and Human Protein Atlas databases, we found that BIRC5 (survivin), BIRC2, and CASP6 (all downregulated by HCQ in Ca9-22 cells) are overexpressed in head and neck squamous cell carcinoma (HNSCC) tissues compared to normal tissues, with expression levels increasing across tumor stages. Notably, Kaplan–Meier survival analyses further indicated that lower expression of these genes is associated with better overall survival in patients with HNSCC. Therefore, HCQ-mediated downregulation of these targets could contribute to improved therapeutic outcomes, mainly patient survival in advanced-stage tumors.

While both CQ and HCQ affected apoptosis-related pathways, significant induction of apoptosis occurred only in Ca9-22 cells, reflecting cell-type-specific susceptibility. The discordance between transcriptional changes and mild phenotypic effects likely reflects the early (24 h) profiling time point.

## 4. Materials and Methods

### 4.1. Cell Culture

To carry out this study, three oral cancer cell lines and one non-oncogenic cell line were selected. Ca9-22 human gingival squamous cell carcinoma line (CVCL_1102), isolated from a 43-year-old Japanese male patient, was purchased from the RIKEN BioResource Research Center (Tsukuba, Ibaraki, Japan). SCC-9 (CRL-1629) and CAL-27 (CRL-2095) human tongue squamous cell carcinoma lines were obtained from ATCC (Manassas, VA, USA). SCC9 cells were isolated from the tongue of a 25-year-old male patient with squamous cell carcinoma, and CAL-27 cells were isolated from tissue taken prior to treatment from a 56-year-old White Caucasian male. GMSM-K, a non-oncogenic human epithelial cell line derived from a 30-week gestational stillborn male fetus, was provided by Dr. Grenier (Laval University). Ca9-22 cells (GC) were routinely grown in RPMI-1640 medium (Gibco, Saint-Laurent, Quebec, QC, Canada, Cat# 31800089), while SCC-9(TC), CAL-27(TC), and GMSM-K cells were maintained in Dulbecco’s modified Eagle’s medium (DMEM)/F-12 (Gibco, cat# 12634010). All cells were authenticated and regularly tested for Mycoplasma contamination. Culture media were supplemented with 10% fetal bovine serum (FBS) (Gibco, cat# A5670801), 0.2% penicillin-streptomycin (Sigma-Aldrich, Oakville, ON, Canada, cat# A2942), and 0.2% fungizone (Sigma-Aldrich, cat# A2942). All cell lines were cultured at 37 °C in a humidified atmosphere containing 5% CO_2_. The culture medium was changed every 2 days until the cells reached 80% confluence.

### 4.2. Treatment Regimen

All cell lines were exposed for 24 h to different concentrations of CQ (Abcam, Cambridge, MA, USA, cat# ab142116) ranging from 10 µM to 250 µM. For HCQ (Abcam, cat# ab120827), the lowest concentration used was 1 µM. CQ and HCQ were solubilized in dimethyl sulfoxide (DMSO) to prepare stock solutions at 50 mM and 100 mM, respectively. Preliminary dose–response assays revealed that HCQ concentrations above 50 µM caused non-selective cytotoxicity in GMSM-K and Cal-27 cells; therefore, analyses were limited to ≤50 µM.

### 4.3. MTT Cell Viability Assay

Cell viability and proliferation were assessed using the MTT assay as previously described [79,80]. Briefly, 3 × 10^5^ cells were seeded per well of 6-well plates, then treated with various concentrations of CQ or HCQ. Following 24 h incubation period, MTT reagent (Sigma-Aldrich, cat# M-2128) at a final concentration of 0.5 mg/mL was added to the cell culture and incubated at 37 °C for 3 h in the dark. The assay principle relies on the ability of viable cells to reduce the yellow tetrazolium salt into purple formazan crystals. The resulting crystals were then dissolved using isopropanol 0.4% HCl, and absorbance was measured at 550 nm using a Bio-Rad iMark™ microplate reader (Biorad, Mississauga, ON, Canada). The half-maximal inhibitory concentration (IC50) was determined for both CQ and HCQ molecules. Four technical replicates and a minimum of six biological replicates were performed.

### 4.4. LDH Cell Cytotoxicity Assay

CQ and HCQ cytotoxicity was assessed using the lactate dehydrogenase (LDH) kit (Roche, Mississauga, ON, Canada, cat#11644793001) as described in our previous works [79,81,82]. Briefly, cells were treated with different concentrations of CQ or HCQ. After 24 h incubation period, supernatants were collected for each condition. Dye and catalyst solutions were then added to the supernatants and incubated for 30 min, allowing the formation of a red formazan product proportional to the amount of LDH released due to plasma membrane damage. 1% of Triton X-100 was used as a positive control. The optical density was measured at 490 nm using a Bio-Rad iMark™ microplate reader (Saint-Laurant, Montreal, QC, Canada). Three technical replicates and at least six biological replicates were performed.

### 4.5. Clonogenic Test

As described by our previous studies [82,83,84], cells were plated in six-well plates at a density of 2 × 10^3^ cells per well and treated with different concentrations of CQ or HCQ for 14 days. After incubation period, the colonies were rinsed with PBS, fixed with cold methanol for 10 min, and stained with a 1% crystal violet solution (Sigma-Aldrich, cat# 548-62-9). Excess stains were removed by washing with sterile water, and images of colony formation were captured using an image scanner. Six biological replicates were performed.

### 4.6. Cell Autophagy Assay

Autophagy was monitored using the Cyto-ID^®^ Red Autophagy Detection Reagent (Immunochemistry Technologies, Davis, CA, USA) by using flow cytometry, as previously described [82,85]. Cells were stained at a 1:5 dilution for 1 h at 37 °C in the dark and analyzed by flow cytometry (FL3-PE). An untreated negative control was included in each run to establish baseline autophagy levels. Flow cytometry analysis was performed using the BD Accuri C6 Plus Flow Cytometer (Mississauga, ON, Canada). Three biological replicates were carried out.

### 4.7. Gene Expression Profiles and Interaction Networks

Ca9-22 and SCC-9 cells were exposed to CQ and HCQ at IC_50_ for 24 h. Afterwards, the cells were trypsinized, and total RNA was isolated and purified using the RNeasy Mini Kit following the manufacturer’s guidelines (Qiagen, Toronto, ON, Canada). RNA concentration and purity were assessed using Nano Drop spectrophotometer (Thermo Fisher, Waltham, MA, USA). A total of 2 µg RNA was reverse transcribed into cDNA using the iScript™ Reverse Transcription Super mix for RT-qPCR (Bio-Rad, cat# 1708841). RT2 Profiler PCR Arrays for apoptosis (Qiagen, Toronto, ON, Canada, cat# PAHS-012ZD) and autophagy (Qiagen, PAHS-084ZF) were used as previously described to investigate the genetic variations associated with CQ and HCQ exposure [85]. The PCR mixture consisted of 1350 μL of SYBR Green Master mix (Bio-Rad, 64204590), 102 μL of cDNA, and 1248 μL of RNase-free water. A total of 25 μL mixture was added to each well of the RT2 Profiler plate, which contains the primers specific to 84 genes involved in apoptosis and autophagy pathways, along with 5 housekeeping genes for normalization. The data acquired through real-time PCR were analyzed using the 2^−ΔΔCT^ method to determine relative gene expression and fold change between untreated and treated cells. Only genes showing at least a two-fold variation relative to controls were considered. CT values were extracted and uploaded to the Qiagen Gene Globe platform (http://www.qiagen.com/geneglobe) (accessed on 3 April 2025) for further data analysis. One representative experiment was carried out. To identify gene interaction networks within the autophagy and apoptosis panels, we employed the Gene MANIA tool (V3.5.3, released on 29 February 2024 (https://genemania.org/) (accessed on 3 April 2025). Functions with low FDR and high coverage were selected to ensure statistical robustness and biological relevance.

### 4.8. Mitochondrial Membrane Potential (∆Ψm) Assay

Ca9-22 and SCC-9 cells were treated with CQ or HCQ for 24 h. Following treatment, cells were trypsinized and washed with sterile PBS. They were then stained with 50 nM of DiOC_2_ (Invitrogen™, Waltham, MA, USA, Cat# D14730) and incubated for 1 h at 37 °C in the dark. After incubation, cells were washed three times to remove excess unbound dye and analyzed using a BD Accuri™ C6 Plus Flow Cytometer (Mississauga, ON, Canada). Green fluorescence was detected via the FL1 (FITC) channel [86,87]. Two to four biological replicates were carried out.

### 4.9. Oxidative Stress: Total ROS, MitoSOX, and GSH

Ca9-22 and SCC-9 cells were treated with CQ or HCQ for 24 h. As described in our previous work, mitochondrial superoxide production was assessed as per the manufacturer’s recommendations using the MitoSOX™ Red Mitochondrial Superoxide Indicator (Invitrogen, Burlington, ON, Canada; Cat# M36008) [87]. The expression of intracellular total ROS was evaluated using the Total ROS Detection Kit (Cat# 9144, ImmunoChemistry Technologies, Bloomington, MN, USA). Intracellular glutathione (GSH) levels were assessed using the ImmunoChemistry Technologies kit by means of the ThioBright™ Green reagent (Cat# 9137, ImmunoChemistry Technologies, Bloomington, MN, USA) [88]. Briefly, all reagents were incubated with the cells for a period between 30 min and 1 h at 37 °C in the dark. Following incubation, cells were washed with sterile PBS to remove excess dye and analyzed by flow cytometry using a BD Accuri™ C6 Plus Flow Cytometer. Fluorescence was detected via the PE and FITC channels. Four biological replicates were carried out.

### 4.10. DNA Damage Analysis (γH2AX Detection)

DNA damage was assessed by flow cytometry through the measurement of phosphorylated histone H2AX (γH2AX, Ser139) [89]. After 24 h treatment, Ca9-22 and SCC-9 cells were collected and washed with sterile PBS. Cells were then fixed with 4% paraformaldehyde for 20 min at room temperature and permeabilized in PBS/10% FBS/0.2% Triton X-100 solution for 30 min. Following a blocking step in PBS/5% FBS solution, cells were incubated overnight at 4 °C with the corresponding primary antibodies diluted at 1/100 in PBS solution containing 10% FBS and 0.05% Triton X-100. Anti-γH2AX (Santa Cruz Biotechnology, Dallas, TX, USA, Ref# sc-51734) primary mouse antibody was used. After two washes with PBS, cells were stained for 1 h at room temperature in the dark with a FITC-conjugated goat anti-mouse IgG secondary antibody (Chemicon International, Temecula, CA, USA Cat# AQ303F) diluted at 1:100 in PBS solution containing 2% FBS. Finally, cells were resuspended in PBS/2% FBS solution and analyzed by flow cytometry using the FL1 channel to quantify p-γH2AX-positive cells. Three biological replicates were carried out.

### 4.11. Cell Apoptosis Assay

Apoptosis induction by CQ and HCQ in oral cancer cells was evaluated using the Annexin V (AnxV)—Propidium Iodide (PI) Apoptosis kit (Biolegend, San Diego, CA, USA) [82,85]. Briefly, after 24 h treatment, Ca9-22 and SCC-9 cells were trypsinized, washed with sterile PBS, and then stained with 10 μL AnxV and 5 μL PI for 15 min at room temperature. Afterwards, 400 μL of AnxV binding buffer was added to each tube, and flow cytometry analysis was performed using the BD Accuri C6 Plus Flow Cytometer. The total events were classified as viable (AnxV−/PI−), early apoptotic (AnxV+/PI−), late apoptotic (AnxV+/PI+), and necrotic cells (AnxV−/PI+). Three biological replicates were carried out.

### 4.12. Statistical Analysis

Statistical analysis was performed using GraphPad Prism 9.1.0 (GraphPad Software, San Diego, CA, USA). All experiments were performed at least in triplicate, and data were expressed as means ± Standard Error of the Mean (SEM). For qPCR array, data analysis was based on the ΔΔC_T_ method with normalization of the raw data to either housekeeping genes. Statistical significance was determined using the one-way Anova test with * *p* < 0.05, ** *p* < 0.01, *** *p* < 0.001 considered as statistically significant.

## 5. Conclusions

CQ and HCQ are proposed as adjuvant or repositioned therapies, not first-line agents, with potential to sensitize chemoresistant tumors to standard drugs by inhibiting autophagy-dependent survival. We acknowledge that the cell lines used are not chemoresistant models, and future work using cisplatin- or 5-FU-resistant derivatives will be essential to confirm whether HCQ can restore chemosensitivity. In conclusion, HCQ selectively targets gingival carcinoma cells by modulating autophagy and mitochondrial stress pathways, supporting its use as a potential adjuvant therapy in oral cancer.

## Data Availability

The original contributions presented in this study are included in the article/Supplementary Materials. Further inquiries can be directed to the corresponding author.

## Supplementary Materials

The following supporting information can be downloaded at: https://www.mdpi.com/article/10.3390/ijms262210994/s1.

## Abbreviations

AnxV	Annexin V
Cal-27	Human tongue carcinoma cell line
Ca9-22	Human gingival squamous cell carcinoma line
CQ	Chloroquine
GMSM-K	Non-oncogenic oral epithelial cells
HCQ	Hydroxychloroquine
LDH	lactate dehydrogenase
MitoSOX	Red Mitochondrial Superoxide
OC	Oral cancer
OSCC	Oral squamous cell carcinoma
PI	Propidium Iodide
SCC-9	Human tongue carcinoma cell line
5-FU	5-fluorouracil
Δψm	Mitochondrial membrane potential
