# Supplementary material for: Repurposing Antimalarials for Oral Cancer: Selective Efficacy of Hydroxychloroquine on Gingival Squamous Cell Carcinoma
Source: Int J Mol Sci. 2025 Nov 13;26(22):10994. doi: 10.3390/ijms262210994 (PMC12652187; doi:10.3390/ijms262210994)
Supplement: Supplementary file 1 [file ijms-26-10994-s001.zip › ijms-3917444-supplementary.pdf]

Table- S1. Differential expression of autophagy-related genes in Ca9-22 cells following CQ and HCQ treatments

Gene	RefSeq (mRNA)	Full name	Fold Change CQ	Fold Change HCQ
WIPI1	NM_017983.6	WD repeat domain phosphoinositide-interacting protein 1	2.06	3.04
ULK1	NM_003565.5	Unc-51 like autophagy activating kinase 1	2.09	-
ATG4C	NM_032852.4	Autophagy related 4C cysteine peptidase	2.09	-
ATG4D	NM_001039012.4	Autophagy related 4D cysteine peptidase	14.74	11.66
MAP1LC3B	NM_022818.3	Microtubule associated protein 1 light chain 3 beta	-	2.37
BCL2L1	NM_138578.5	B-cell CLL/lymphoma 2 like 1 (BCL-X)	-	2.70
MAP1LC3A	NM_022817.3	Microtubule associated protein 1 light chain 3 alpha	-	2.37
HSP8	NM_007075.3	Heat shock protein family A (Hsp70) member 8	-	-2.52
PIK3R4	NM_030957.5	Phosphoinositide-3-kinase regulatory subunit 4	-	-2.32
NFKB1	NM_001165412.3	Nuclear factor kappa B subunit 1	-	-2.06
LAMP1	NM_005561.4	Lysosomal associated membrane protein 1	-	-2.58
IRGM	NM_001297412.2	Immunity-related GTPase M	-2.28	-3.70
HTT	NM_002111.10	Huntingtin	-2.01	-3.00
HSP90AA1	NM_007355.6	Heat shock protein 90 alpha family class A member 1	-	-2.80
AKT1	NM_005163.3	AKT serine/threonine kinase 1	-2.75	-2.37
GAA	NM_000152.5	Glucosidase alpha, acid	-2.73	-2.67
AMBRA1	NM_001199420.2	Autophagy/beclin 1 regulator 1	-2.19	-2.37
EIF4G1	NM_001972.3	Eukaryotic translation initiation factor 4 gamma 1	-2.26	-3.04
CTSD	NM_001909.5	Cathepsin D	-2.41	-2.49
CDKN1B	NM_004064.4	Cyclin dependent kinase inhibitor 1B (p27, Kip1)	-2.29	-2.20
BID	NM_032982.3	BH3 interacting domain death agonist	-3.13	-3.02
BAX	NM_138761.2	BCL2 associated X protein		-2.17
ATG9A	NM_001024647.4	Autophagy related 9A	-3.50	-4.30
ATG7	NM_001320884.3	Autophagy related 7	-2.13	-2.24
ATG16L2	NM_001130222.2	Autophagy related 16 like 2	-2.41	-2.21
FADD	NM_001293615.2	Fas associated via death domain	-2.03	-

Table- S1. Effect of CQ and HCQ on the expression profile of autophagy-related genes in Ca9-22 cells. This table presents the RefSeq accession numbers and full gene names corresponding to the autophagy-related genes that were modulated in response to 50 μM CQ and HCQ treatments in Ca9-22 gingival carcinoma cells. A total of 84 autophagy-related genes were analyzed using a quantitative PCR array. Only genes exhibiting a fold change of ±2 or more relative to untreated controls were included in the analysis.

Table-S2. Differential expression of autophagy-related genes in SCC-9 cells following CQ and HCQ treatments

Gene	RefSeq (mRNA)	Full name	Fold Change CQ	Fold Change HCQ
AKT1	NM_005163.3	AKT serine/threonine kinase 1	2.94	4.96
IFNG	NM_000619.4	Interferon gamma	19.24	-
MAPK8	NM_002750.5	Mitogen-activated protein kinase 8 (JNK1)	7.04	22.01
MAPK14	NM_001315.8	Mitogen-activated protein kinase 14 (p38α)	6.26	12.64
MAP1LC3B	NM_022818.3	Microtubule-associated proteins 1A/1B light chain 3B	27.02	-4.23
MAP1LC3A	NM_022817.3	Microtubule-associated proteins 1A/1B light chain 3A	5.80	124.50
LAMP1	NM_005561.4	Lysosomal associated membrane protein 1	2.36	-
INS	NM_000207.3	Insulin	-	4.56
IGF1	NM_000618.3	Insulin-like growth factor 1	20.20	-
HTT	NM_002111.10	Huntingtin	8.32	-
NFKB1	NM_001165412.3	Nuclear factor kappa B subunit 1 (p105)	2.36	99.04
HSP90AA1	NM_007355.6	Heat shock protein 90 alpha family class A member 1	-	5.31
HGS	NM_015277.4	Hepatocyte growth factor-regulated tyrosine kinase substrate	-	1530.73
HDAC6	NM_007297.4	Histone deacetylase 6	6.62	9.32
HDAC1	NM_004964.4	Histone deacetylase 1	4.55	-
GABARAPL2	NM_001256485.1	GABA type A receptor-associated protein-like 2	5.26	-
GABARAPL1	NM_016330.4	GABA type A receptor-associated protein-like 1	7.65	108.38
AMBRA1	NM_001199420.2	Autophagy/beclin 1 regulator 1	20.34	6.19
MTOR	NM_004958.4	Mechanistic target of rapamycin kinase	3.47	-
NPC1	NM_000271.5	Niemann-Pick disease, type C1 protein	-	3.36
FAS	NM_000043.6	Fas cell surface death receptor	3.75	-3.29
SNCA	NM_000345.4	Synuclein alpha	3.04	183.55
UVRAG	NM_004543.3	UV radiation resistance-associated gene	7.60	109.14
ULK2	NM_001001517.3	Unc-51 like autophagy activating kinase 2	4.65	2.36
ULK1	NM_003565.5	Unc-51 like autophagy activating kinase 1	10.60	25.28
TP53	NM_000546.6	Tumor protein p53	14.99	7.84

TNF	NM_000594.4	Tumor necrosis factor	10.53	191.34
TMEM74	NM_207457.2	Transmembrane protein 74	12.61	5.21
TGFB1	NM_000660.7	Transforming growth factor beta 1	2.56	6.41
RPS6KB1	NM_002730.6	Ribosomal protein S6 kinase B1	5.16	-
PIK3C3	NM_002647.1	Phosphatidylinositol 3-kinase catalytic subunit type 3	4.98	-5.39
RGS19	NM_005857.5	Regulator of G-protein signaling 19	17.34	86.22
RB1	NM_000321.2	RB transcriptional corepressor 1	3.52	-3.43
RAB24	NM_006881.4	RAB24, member RAS oncogene family	10.97	76.11
PTEN	NM_000314.9	Phosphatase and tensin homolog	5.49	-6.73
PRKAA1	NM_006252.4	Protein kinase AMP-activated catalytic subunit alpha 1	4.68	14.62
PIK3R4	NM_030957.5	Phosphoinositide-3-kinase regulatory subunit 4	3.55	10.48
PIK3CG	NM_000855.4	Phosphoinositide-3-kinase catalytic subunit gamma	24.69	56.10
GAA	NM_000152.5	Glucosidase alpha, acid	12.87	464.65
WIPI1	NM_017983.4	WD repeat domain phosphoinositide-interacting protein 1	2.74	421.68
FADD	NM_001293615.2	Fas associated via death domain	4.84	16.45
ATG4C	NM_032852.4	Autophagy related 4C cysteine peptidase	6.17	6.11
BAK1	NM_001188.3	BCL2 antagonist/killer 1	7.44	8.94
BAD	NM_004322.3	BCL2 associated agonist of cell death	3.83	15.67
ATG9B	NM_001145086.2	Autophagy related 9B	10.45	40.50
ATG9A	NM_001024647.4	Autophagy related 9A	17.46	-
ATG7	NM_001320884.3	Autophagy related 7	5.60	-
ATG5	NM_004849.4	Autophagy related 5	3.83	-
ATG4D	NM_001039012.4	Autophagy related 4D cysteine peptidase	8.85	136.24
ATG4B	NM_001127290.4	Autophagy related 4B cysteine peptidase	10.75	5.17
BCL2L1	NM_138578.5	BCL2-like 1	10.38	73.01
ATG4A	NM_006411.4	Autophagy related 4A cysteine peptidase	4.19	6.15
ATG3	NM_004849.5	Autophagy related 3	8.85	5.82
ATG16L2	NM_001130222.2	Autophagy related 16 like 2	13.99	3.97
ATG16L1	NM_004722.3	Autophagy related 16 like 1	4.10	-
ATG12	NM_004707.4	Autophagy related 12	6.30	6.36
ATG10	NM_006713.4	Autophagy related 10	7.92	2.66
APP	NM_000484.4	Amyloid beta precursor protein	-	6.96
ESR1	NM_000125.5	Estrogen receptor 1	6.95	9.71

BAX	NM_138761.2	BCL2 associated X protein	2.92	-8.63
BECN1	NM_003766.4	Beclin 1	3.20	3.18
DRAM1	NM_018292.3	DNA damage regulated autophagy modulator 1	2.39	12.64
EIF4G1	NM_001972.3	Eukaryotic translation initiation factor 4 gamma 1	2.82	-3.34
CASP3	NM_004346.3	Caspase 3	-	6.19
CASP8	NM_001228.3	Caspase 8	4.98	2.87
CDKN1B	NM_004064.4	Cyclin dependent kinase inhibitor 1B	4.52	-
CDKN2A	NM_000077.4	Cyclin dependent kinase inhibitor 2A	20.91	54.95
CLN3	NM_001042426.2	CLN3, Batten disease gene	3.57	31.56
CTSB	NM_001908.3	Cathepsin B	5.41	10.78
CTSD	NM_001909.5	Cathepsin D	3.88	2.58
CTSS	NM_012168.3	Cathepsin S	2.61	-3.73
BID	NM_032982.3	BH3 interacting domain death agonist	3.42	537.45
EIF2AK3	NM_004836.3	Eukaryotic translation initiation factor 2 alpha kinase 3	5.41	4.99
DRAM2	NM_018293.3	DNA damage regulated autophagy modulator 2	6.80	2.91
CXCR4	NM_001008540.3	C-X-C motif chemokine receptor 4	22.25	-
DAPK1	NM_001323051.1	Death-associated protein kinase 1	15.74	-
SQSTM1	NM_003900.5	Sequestosome 1	-	-4.69
TNFSF10	NM_024420.4	TNF superfamily member 10	-2.19	-25.28
TGM2	NM_001145673.3	Transglutaminase 2	-	-3.10
HSPA8	NM_006597.4	Heat shock 70kDa protein 8	-	-133.44
BNIP3	NM_004052.4	BCL2 interacting protein 3	-	-33.82
GABARAP	NM_005650.4	GABA type A receptor-associated protein	-2.15	-3.05

Table-S2. **Effect of CQ and HCQ on the expression profile of autophagy-related genes in SCC-9 cells.** This table presents the RefSeq accession numbers and full gene names corresponding to the autophagy-related genes that were modulated in response to 50 μM CQ and HCQ treatments in SCC-9 cells. A total of 84 autophagy-related genes were analyzed using a quantitative PCR array. Only genes exhibiting a fold change of ±2 or more relative to untreated controls were included in the analysis.

Table-S3. Differential expression of apoptosis-related genes in Ca9-22 cells following CQ and HCQ treatments

Gene	RefSeq (mRNA)	Full name	Fold Change CQ	Fold Change HCQ
BAK1	NM_001188.3	BCL2 antagonist/killer 1	2.04	-
BCL2A1	NM_004049.4	BCL2 related protein A1	2.61	-
BIK	NM_001197.4	BCL2 interacting killer	-	2.05
CYCS	NM_018947.4	Cytochrome c, somatic	4.16	-
DAPK1	NM_001323051.1	Death-associated protein kinase 1	4.22	-2.71
RIPK2	NM_003821.4	Receptor interacting serine/threonine kinase 2	3.31	-
TNFRSF11B	NM_002546.5	TNF receptor superfamily member 11b (osteoprotegerin)	2.52	-8.67
APAF1	NM_001160.4	Apoptotic peptidase activating factor 1	-	-2.23
BIRC2	NM_001165.5	Baculoviral IAP repeat containing 2 (cIAP1)	-	-3.22
BIRC5	NM_001012271.2	Baculoviral IAP repeat containing 5 (survivin)	-	-2.65
CASP2	NM_032982.3	Caspase 2	-2.35	-2.80
CASP6	NM_001226.4	Caspase 6	-	-2.98
TRADD	NM_003789.4	TNFRSF1A associated via death domain	-	-2.51
TRAF2	NM_021138.4	TNF receptor associated factor 2	-	-2.23
XIAP	NM_001167.3	X-linked inhibitor of apoptosis	-4.80	-

Table-S3. Effect of CQ and HCQ on the expression profile of apoptosis-related genes in Ca9-22 cells. This table presents the RefSeq accession numbers and full gene names corresponding to the apoptosis-related genes that were modulated in response to 50 μM CQ and 10 μM HCQ treatments in Ca9-22 gingival carcinoma cells. A total of 84 apoptosis-related genes were analyzed using a quantitative PCR array. Only genes exhibiting a fold change of ±2 or more relative to untreated controls were included in the analysis.

Table-S4. Differential expression of apoptosis-related genes in Ca9-22 cells following CQ and HCQ treatments

AIFM1	**NM_004208.4**	**Apoptosis inducing factor mitochondria associated 1**	**3.14**	
NAIP	NM_004536.3	NLR family apoptosis inhibitory protein	15.58	
CYCS	NM_018947.4	Cytochrome c, somatic	61.05	2.90
DAPK1	NM_001323051.1	Death-associated protein kinase 1	-	2.53
DFFA	NM_004402.4	DNA fragmentation factor subunit alpha	2.30	
FADD	NM_001293615.2	Fas associated via death domain	4.57	
FAS	NM_000043.6	Fas cell surface death receptor	3.79	
FASLG	NM_000639.2	Fas ligand	14.74	
GADD45A	NM_001924.4	Growth arrest and DNA damage inducible alpha	2.40	
HRK	NM_003822.5	Harakiri, BCL2 interacting protein	39.45	
IGF1R	NM_000875.4	Insulin like growth factor 1 receptor	3.14	
LTA	NM_000595.4	Lymphotoxin alpha	18.03	
MCL1	NM_021960.5	MCL1 apoptosis regulator	8.41	
NOD1	NM_006092.4	Nucleotide binding oligomerization domain containing 1	14.05	
AKT1	NM_005163.3	AKT serine/threonine kinase 1	2.93	
RIPK2	NM_003821.4	Receptor interacting serine/threonine kinase 2	5.04	
TNF	NM_000594.4	Tumor necrosis factor	9.73	
TNFRSF10A	NM_003842.4	TNF receptor superfamily member 10A	12.75	
TNFRSF10B	NM_003842.5	TNF receptor superfamily member 10B	5.47	
TNFRSF11B	NM_002546.5	TNF receptor superfamily member 11b	3.04	
TNFRSF1B	NM_001066.3	TNF receptor superfamily member 1B	17.05	
TNFRSF9	NM_003811.4	TNF receptor superfamily member 9	11.49	
TP53BP2	NM_005426.4	Tumor protein p53 binding protein 2	4.06	
TRADD	NM_003789.4	TNFRSF1A associated via death domain	8.29	
TRAF2	NM_021138.4	TNF receptor associated factor 2	11.89	
TRAF3	NM_145725.3	TNF receptor associated factor 3	3.39	
CRADD	NM_003805.4	CASP2 and RIPK1 domain containing adaptor with death domain	8.47	
CIDEB	NM_001007233.2	Cell death-inducing DFFA-like effector b	7.27	
CIDEA	NM_014426.4	Cell death-inducing DFFA-like effector a	21.89	
BIRC5	NM_001012271.2	Baculoviral IAP repeat containing 5	5.00	
APAF1	NM_001160.4	Apoptotic peptidase activating factor 1	8.41	
BAD	NM_004322.3	BCL2 associated agonist of cell death	3.37	
BAG1	NM_004323.4	BCL2 associated athanogene 1	7.22	
BAK1	NM_001188.3	BCL2 antagonist/killer 1	5.04	
BAX	NM_138761.2	BCL2 associated X protein	2.77	

BCL10	NM_003921.4	B cell leukemia/lymphoma 10	4.20	
BCL2A1	NM_004049.4	BCL2 related protein A1	8.71	
BCL2L1	NM_138578.5	BCL2 like 1	7.07	
BCL2L11	NM_138621.4	BCL2 like 11	8.29	
BID	NM_032982.3	BH3 interacting domain death agonist	3.66	
CFLAR	NM_003879.5	CASP8 and FADD like apoptosis regulator	5.14	
BIRC3	NM_001165.5	Baculoviral IAP repeat containing 3	3.23	
BIRC6	NM_016252.3	Baculoviral IAP repeat containing 6	6.64	-3.84
BNIP2	NM_004327.4	BCL2 interacting protein 2	3.12	
BRAF	NM_004333.6	B-Raf proto-oncogene	12.75	
CASP1	NM_033292.4	Caspase 1	3.74	
CASP10	NM_001230.4	Caspase 10	10.79	
CASP2	NM_032982.3	Caspase 2	6.24	
CASP4	NM_001225.4	Caspase 4	2.32	
CASP8	NM_001228.3	Caspase 8	4.09	
CASP9	NM_001229.4	Caspase 9	5.86	
CD40	NM_001250.4	CD40 molecule	18.03	
CD70	NM_001252.4	CD70 molecule	10.21	
XIAP	NM_001167.3	X-linked inhibitor of apoptosis	4.03	
BCL2	NM_000633.3	BCL2 apoptosis regulator	-	-3.37
TNFSF10	NM_024420.4	TNF superfamily member 10	-2.01	

Table-S4. **Effect of CQ and HCQ on the expression profile of apoptosis-related genes in SCC-9 cells**. This table presents the RefSeq accession numbers and full gene names corresponding to the apoptosis-related genes that were modulated in response to 50 μM CQ and HCQ treatments in SCC-9 cells. A total of 84 apoptosis-related genes were analyzed using a quantitative PCR array.
